# Tachycardia detection in ICDs by Boston Scientific: Algorithms, pearls, and pitfalls

**DOI:** 10.1007/s00399-016-0454-2

**Published:** 2016-09-07

**Authors:** Norbert Zanker, Diane Schuster, James Gilkerson, Kenneth Stein

**Affiliations:** Guidant Europe NV, Boston Scientific, Green Square, Lambroekstraat 5D, 1831 Diegem, Belgium

**Keywords:** Sensing, Detection, Discrimination, Delivery, therapy, Recording, episode history, Wahrnehmung, Detektion, Diskriminierung, Therapieabgabe, Aufzeichnung der Episodenhistorie

## Abstract

**Aim:**

The aim of this study was to summarize how implantable cardioverter defibrillators (ICDs) by Boston Scientific sense, detect, discriminate rhythms, and classify episodes.

**Methods:**

Modern devices include multiple programming selections, diagnostic features, therapy options, memory functions, and device-related history features. Device operation includes logical steps from sensing, detection, discrimination, therapy delivery to history recording. The program is designed to facilitate the application of the device algorithms to the individual patient’s clinical needs. Features and functions described in this article represent a selective excerpt by the authors from Boston Scientific publicly available product resources.

**Conclusion:**

Programming of ICDs may affect patient outcomes. Patient-adapted and optimized programming requires understanding of device operation and concepts.

## Zusammenfassung

**Ziel:**

Ziel dieser Arbeit ist die Zusammenfassung der Arrhythmiewahrnehmung, Detektion, Diskriminierung und Episodenklassifizierung bei ICDs der Fa. Boston-Scientific.

**Methoden:**

Moderne Aggregate beinhalten multiple Programmieroptionen, diagnostische Funktionen, Therapieoptionen, Speicherfunktionen und Funktionen zur aggregatbezogenen Historie. Die Aggregatfunktion beinhaltet logische Schritte von der Wahrnehmung, Detektion, Diskrimierung, Therapieabgabe bis zur Speicherung der Historie. Das Programmiergerät ist so konzipiert, dass es die Anwendung von Gerätealgorithmen zur individuellen Patientenprogrammierung erleichtert. Eigenschaften und Funktionen, die in diesem Manuskript beschrieben sind, stellen eine durch die Autoren getroffene Auswahl aus den von Boston-Scientific öffentlich zugänglichen Produktinformationen dar.

**Schlussfolgerung:**

Die Programmierung von ICDs kann Einfluss auf den Therapieerfolg für den Patienten nehmen. Eine individuelle und patientenbezogene Programmierung erfordert das Verständnis von Aggregatfunktionen und Konzepten.

## Introduction

Modern implantable cardioverter defibrillator (ICD) devices include multiple programming selections, diagnostic features, therapy options, memory functions, and device-related history features. Patient-adapted and optimized programming requires an understanding of the device operation and its concepts. Device operation includes logical steps from sensing, detection, discrimination, and therapy delivery to history recording. The program is designed to facilitate the application of the device algorithms to an individual patient’s clinical needs. Most patients present with a ventricular tachyarrhythmia (VT) that is not immediately syncopal. However, some patients have complex arrhythmia portfolios as well as arrhythmias that are not amenable to device therapy, such as atrial fibrillation. These patients require more complex programming. Hence, it is important to consider applying only those features necessary for an individual patient. The therapy requirements for the various arrhythmias determine the number of therapy zones. These, in conjunction with the hemodynamic tolerance of each arrhythmia, determine the therapy to be programmed as well the time to therapy delivery. Rhythm discriminators are intended to be used when a patient presents with treatable and nontreatable rhythms in same therapy zone. Functions and algorithms pertinent to tachycardia detection and discrimination are outlined in this review. They are also explained in more detail in the Boston Scientific ICD reference guide and technical manual [1]. The Boston Scientific S‑ICD concept and algorithms are different from transvenous ICDs/cardiac resynchronization therapy defibrillators (CRT-Ds) and are not included in this manuscript.

## Sensing

Heart rate sensing is critical to all detection decisions. The ICD relies on the following to determine cardiac cycle length: a) bipolar electrodes in the atrium and ventricles; b) digital circuits intended to amplify, filter, and rectify the incoming electrogram (EGM) to process only appropriate waveforms, e. g., without T waves; c) automatic gain-controlled sensing circuit for rate sensing. This circuit ensures proper rate sensing by compensating for changing or diminished signal amplitudes. In CRT-D/CRT-pacemaker (P) devices involving an active left ventricular (LV) lead, multiple programmable LV pace and sense configurations are available.

## Calculating rates and refractory periods

The ICD evaluates rate on an interval-by-interval basis. Following a sensed cardiac depolarization, a cycle length is measured and compared with the programmed detection parameters. The pulse generator uses refractory periods following paced and sensed intrinsic events because the system paces and senses from the *same* electrodes – unlike EP systems – which have dedicated pace and sense electrodes. Events that fall within these periods are ignored for detection purposes. The refractory periods, together with noise windows, may prevent the sensing of nonphysiologic signals and the potential delivery of unwanted therapy (Table 1).

**Table 1:** Nonprogrammable refractory periods

85 ms	Atrial refractory period following an atrial sensed event
150 ms	Atrial refractory period following an atrial pace in DDD(R) and DDI(R) modes
135 ms	Right ventricular refractory period following a right ventricular sensed event
135 ms	Refractory period following a capacitor charge (sensing is ignored in all chambers)
500 ms	Refractory period following shock delivery (sensing is ignored in all chambers)

The ICD compares each sensed right ventricular cardiac cycle interval against the programmed VT rate threshold.

## Therapy zones

A VT zone is a range of heart rates defined by at least one programmed VT rate threshold (Table 2). Zones are created by programming from 1 to 3 VT zones, each of which can be treated by a separate therapy prescription.

**Table 2:** Nominal values for ventricular rate threshold configuration

Ventricular zone configuration	VT-1 zone	VT zone	VF zone
1 Zone	–	–	200 min−1
2 Zones	–	160 min−1	200 min−1
3 Zones	140 min−1	160 min−1	200 min−1

*VF* ventricular fibrillation, *VT* ventricular tachyarrhythmia

The programmer aids in selecting device parameters by including interactive limits and providing alternatives in the graphic user interface to avoid programming of non-executable combinations.

The design concept of current Boston Scientific Corporation (BSC) defibrillators remains unchanged since the Cardiac Pacemakers Inc. (CPI) design. However, the system has been modified to incorporate engineering advances in computer and hardware technology.

## Ventricular detection windows

Appropriate therapy delivery is dependent upon accurately classifying a patient’s rhythm. To ensure that appropriate therapy is delivered, the ICD employs detection windows to differentiate tachycardias. Each ventricular zone has a detection window that consists of the 10 most recent right ventricular R–R intervals measured by the system. As each new interval is measured, it is compared with each zone’s programmed rate threshold and classified as either fast or slow (i. e., above or below the rate threshold) in each detection window. The pulse generator prepares for a potential episode when it counts 3 consecutive fast intervals. The detection window is satisfied and an episode is declared when 8 out of 10 fast intervals are counted. The detection window will remain satisfied as long as 6 of 10 intervals remain classified as fast. If the number of fast intervals falls below 6, the zone’s detection window is no longer satisfied. The zone’s detection window will only become re-satisfied when 8 of 10 intervals are again classified as fast. Because the rate threshold in the higher zones must be programmed to a value greater than the rate threshold in lower zones, an interval classified as fast in a higher window would also be classified as fast in any lower windows.

## Duration parameter

**Fig. 1:**
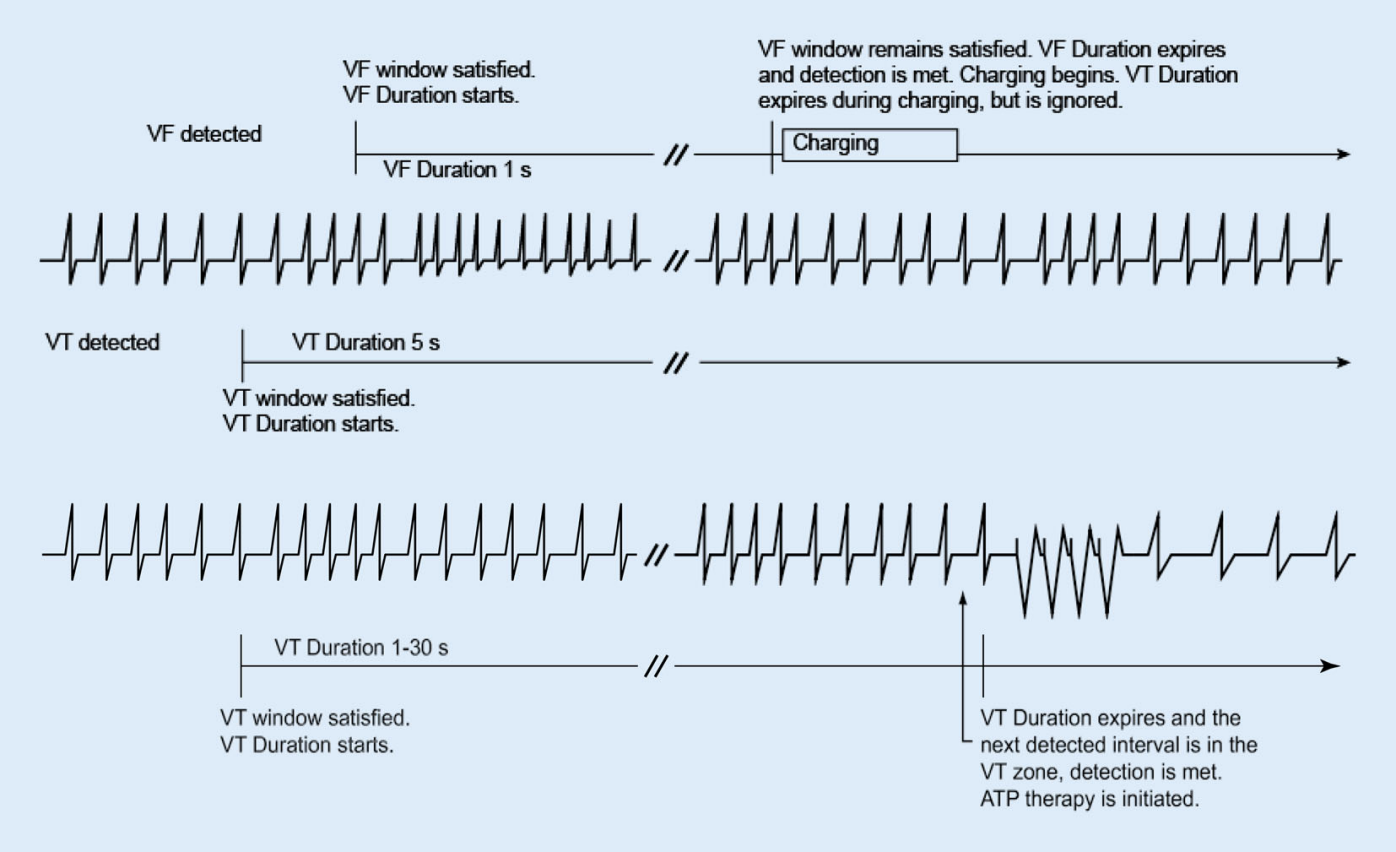
Ventricular tachyarrhythmia (*VT*) detection window remains satisfied during VT duration. *ATP* antitachycardia pacing therapy, *VF* ventricular fibrillation

The *duration* parameter is the timer that measures the numbers of seconds in each zone that a rhythm must be sustained before therapy is delivered. The duration timer begins when its respective zone’s detection window is satisfied (Fig. 1). The programmed duration time is checked following every cardiac cycle to determine whether it has expired. If the last detected interval is in the zone when its duration time expires, detection is considered met for that zone and antitachycardia pacing therapy (ATP) or high-voltage charging is initiated (assuming no programmed detection enhancements inhibit therapy delivery). If at any point during duration a zone’s detection window detects fewer than 6 of 10 fast intervals, that zone’s duration is reset to 0. Duration will start again only if the detection window becomes re-satisfied in that zone.

**Fig. 2:**
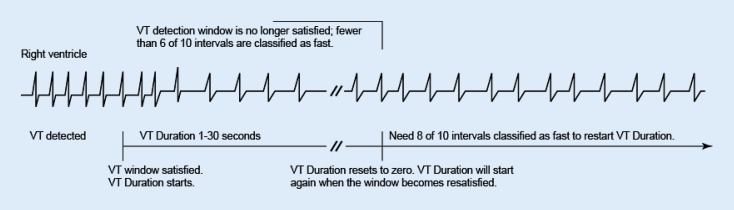
Ventricular tachyarrhythmia (*VT*) detection window is no longer satisfied; fewer than 6 of 10 intervals are classified as fast

Ventricular duration timer. Duration starts when a window becomes satisfied and continues to elapse as long as the ventricular detection window remains satisfied. Detection is met when duration expires and the last detected interval is in the same ventricular zone (Fig. 1). If fewer than 6 of 10 intervals are classified as fast, VT duration resets to zero (Fig. 2).

## Duration in a multizone configuration

**Fig. 3:**
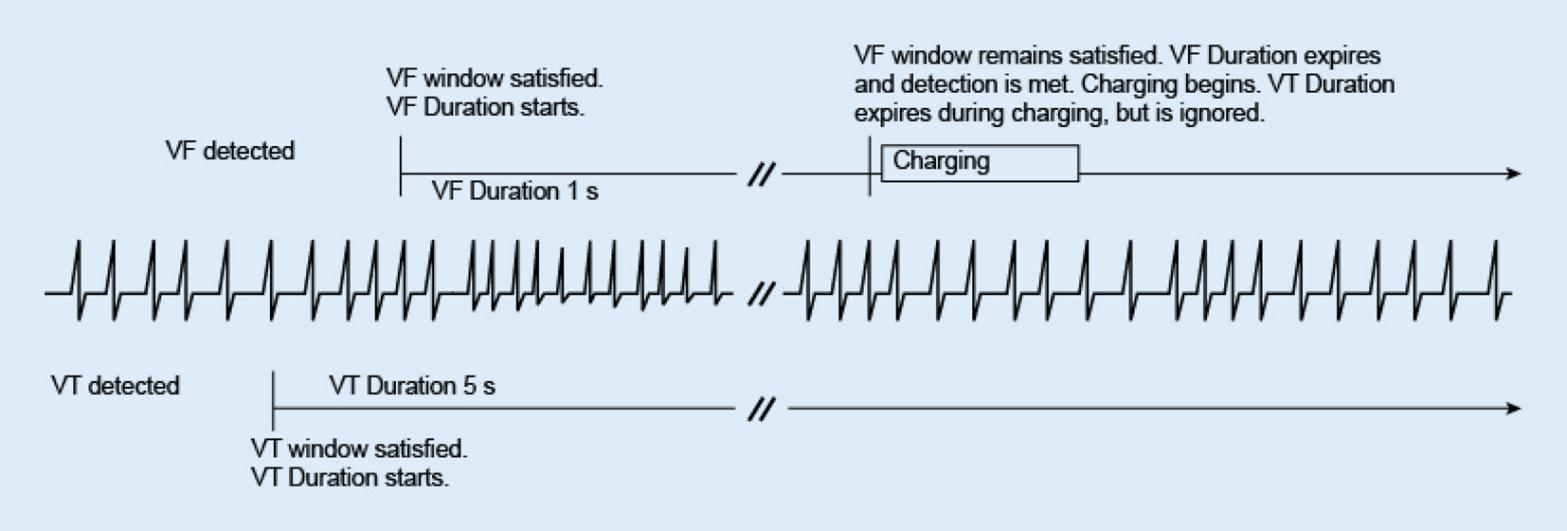
Interaction of ventricular duration, 2‑zone configuration, charging. *VF* ventricular fibrillation, *VT* ventricular tachyarrhythmia

Duration timers run independently of each other within their respective ventricular zones. If the arrhythmia is detected in the highest zone, that zone’s duration timer takes precedence over the lower zones’ timers; the lower zones’ duration timers continue to elapse but are ignored while the higher zone’s duration timer runs. If the higher zone’s duration expires and detection is met, therapy for that zone will be initiated regardless of whether the lower zones’ duration timers have expired (Fig. 3). If the higher zone’s detection window does not remain satisfied, then the duration timers for the lower ventricular zones are no longer ignored. Programmed therapy for lower ventricular zones will be initiated when a lower ventricular zone’s duration is met and no higher ventricular zone’s window is satisfied.

## Ventricular detection enhancements

There are two programmable suites of detection enhancements. Interval-based onset/stability algorithms and vector-based RhythmID® are available in all transvenous ICDs and CRT-Ds by Boston Scientific.

## Design concepts

The design concept of the ICD is to deliver antitachycardia therapy (ATP/shock) when the heart rate exceeds the lowest programmed rate zone, e. g., rate-only programming. With detection enhancements, active therapy will be delivered unless the detection enhancements determine that the rhythm does not meet treatment criteria. If concomitant arrhythmias in the atrium and ventricle are present, the ventricular algorithms will have priority and determine if therapy delivery is appropriate. The determination of all detection enhancement values is made at the end of duration to decide if therapy is inhibited or executed. In devices with an atrial and ventricular lead, when the ventricle is fast and the atrium is slow, thus indicating AV dissociation, the device will proceed with ventricular therapy (V >A). In dual-chamber devices the primary goal was to eliminate ventricular therapy delivered for atrial fibrillation. Therefore the onset/stability algorithms can be used if the patient’s primary untreatable rhythm is atrial fibrillation. Vector correlation may be used to discriminate sinus tachycardia from ventricular tachycardia. When specific rhythm discrimination is selected, you can modify the values for the detection enhancements that are suitable for discriminating that rhythm. The individual detection enhancement parameters that are available depend on the number of tachy zones that are programmed 3, 2, or 1. It should be noted that different manufacturers use similar terminology to describe device functions, but the devices operate differently. Do not assume that values for a given algorithm are equivalent from company to company.

## Onset

The onset enhancement differentiates physiologic sinus tachycardias, which typically begin slowly, from pathologic tachycardias, which typically begin abruptly. It measures the rate of transition in the ventricular rhythm from slow rates to tachycardia. If the rate increase is gradual, (thus physiologic) it enables the device to inhibit ventricular therapy.

## Stability

**Fig. 4:**
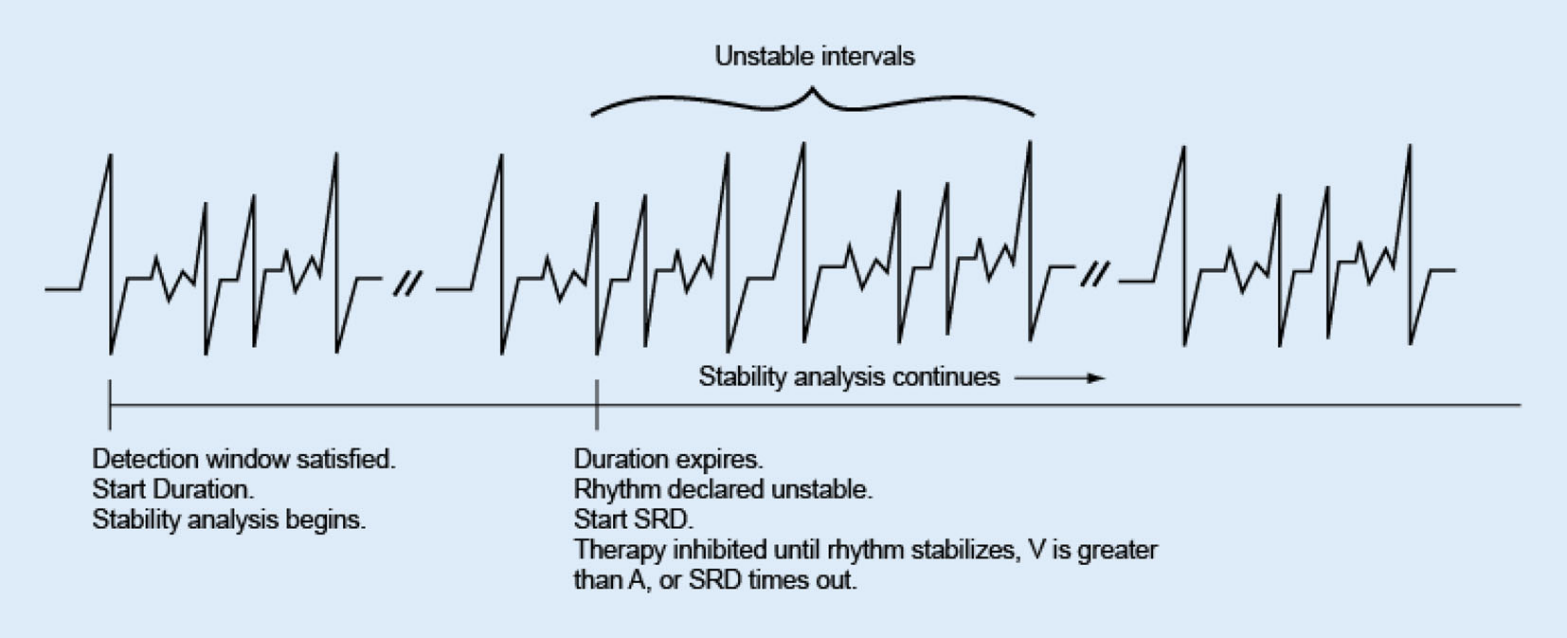
Stability evaluation when duration expires. *SRD* sustained rate duration

Stability enhancement is used to evaluate the variance of ventricular cycle length to determine the regularity of the rhythm. The calculation uses a weighted average during detection. Atrial fibrillation typically results in unstable ventricular rhythms whose rate exceeds the lowest rate threshold and should not be treated. If a rhythm is declared stable (VT) when duration expires, programmed therapy will be delivered. If the rhythm is declared unstable, ventricular therapy will be inhibited (Fig. 4).

At the end of initial duration, if a tachycardia is declared unstable and ventricular therapy is inhibited, the pulse generator continues to evaluate for stability on each new detected interval until the episode is declared over.

Since dual-chamber devices provide knowledge of the atrial activity, the ventricular stability value may be programmed to a more aggressive value, i. e., 20 ms instead of the single-chamber value of 30 ms.

## Sustained rate duration

Sustained is a programmable additional timer and when programmed on, the sustained rate duration (SRD) becomes active only at the end of duration when a “inhibit decision” is made. If at any time during SRD the underlying rhythm changes from inhibit to treat condition, therapy will be initiated. If SRD times out and the patient’s underlying rhythm remains in the rate zone, therapy will be initiated (Table 3).

**Table 3:** Atrial fibrillation rate threshold, stability, and onset combinations and resulting ventricular therapy

Detected ventricular rhythm^a^	Therapy decision^b^
Gradual, unstable, A > AFib rate threshold	Inhibit
Gradual, unstable, A < AFib rate threshold	Inhibit
Sudden, unstable, A > AFib rate threshold	Inhibit
Sudden, unstable, A < AFib rate threshold	Treat^c^
Gradual, stable, A > AFib rate threshold	Treat
Gradual, stable, A < AFib rate threshold	Inhibit
Sudden, stable, A > AFib rate threshold	Treat
Sudden, stable, A < AFib rate threshold	Treat

^a^If the detected ventricular rhythm changes, then the appropriate, corresponding row in the table is evaluated.

^b^Decisions to inhibit can be overridden by V > A or expiration of SRD.

^c^If V rate > A rate is programmed to on and is false, ventricular therapy will be inhibited because the rhythm is unstable.

*AFib* atrial fibrillation

If V rate > A rate is programmed to on and is true, it will take precedence over all inhibitor enhancements.

## Vector timing and correlation

Vector timing and correlation compares EGM signals from an ongoing arrhythmia with a stored reference template of the EGM signals of the patient’s normal sinus rhythm (NSR). Rhythms that are not correlated to the stored reference template are classified as VT. Rhythms that are correlated with the stored reference template are classified as supraventricular tachycardia (SVT). Rhythm ID uses this classification during initial detection to make a decision to treat or to inhibit therapy (Table 4).

**Table 4:** Atrial fibrillation rate threshold, stability, and vector timing and correlation combinations along with resulting therapy decision if atrial tachyarrhythmia discrimination is programmed to on

Detected ventricular rhythm^ a, b, c^	Therapy decision^d^
Correlated, unstable, A > AFib rate threshold	Inhibit
Correlated, unstable, A < AFib rate threshold	Inhibit
Uncorrelated, unstable, A > AFib rate threshold	Inhibit
Uncorrelated, unstable, A < AFib rate threshold	Treat
Correlated, stable, A > AFib rate threshold	Inhibit
Correlated, stable, A < AFib rate threshold	Inhibit
Uncorrelated, stable, A > AFib rate threshold	Treat
Uncorrelated, stable, A < AFib rate threshold	Treat

^a^If the detected ventricular rhythm changes, then the appropriate, corresponding row in the table is evaluated.

^b^If a Rhythm ID reference template is not available, the detected ventricular rhythm is considered to be uncorrelated.

^c^For post-shock detection (if enabled), vector timing and correlation is considered to be uncorrelated.

^d^Decisions to inhibit can be overridden by V > A or expiration of SRD.

## Arrhythmia discrimination discussion

Supraventricular arrhythmias may lead to inappropriate therapy delivery in ICD recipients. The RIGHT Trial was the first randomized head-to-head comparison of ICD discrimination algorithms sponsored by Boston Scientific and published by Gold [2] in 2012. A two-zone programming was required with a VT zone of 150 bpm and a VF zone of 200 bpm including discriminators. In this study, there were more episodes of atrial fibrillation, atrial flutter, and sinus tachycardia receiving inappropriate therapy with VITALITY 2 devices, whereas inappropriate therapy was more common among Medtronic devices for artifact or atrial tachycardia [2]. The increase in the risk of inappropriate therapy with VITALITY 2 devices was significantly greater for single-chamber but not dual-chamber devices. Inappropriate therapies, and differences in performance, may be reduced with the use of a rate cut-off above 175 bpm [2].

As already anticipated by Gold, there are now newer publications listed in the “Device programming considerations” section of this manuscript that show a reduction of inappropriate therapies by using different settings for VT and VF zones including discriminators.

## Programming the RhythmID® with RhythmMatch™ threshold

**Fig. 5:**
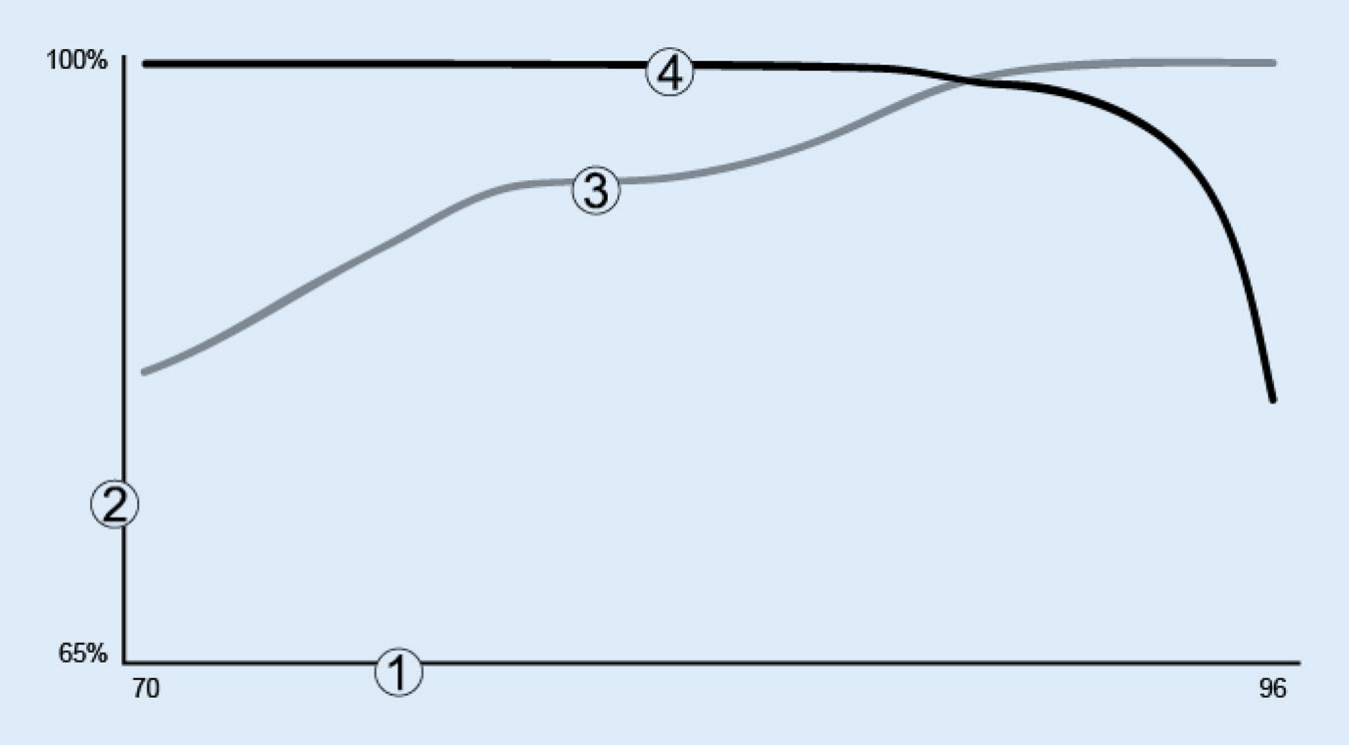
Relationship between sensitivity and specificity using RhythmMatch threshold (*1*). Programmed RhythmMatch threshold (%) (*2*), sensitivity or specificity percentage (*3*), sensitivity to ventricular tachycardia (VT; *4*), specificity for supraventricular tachycardia (SVT)

The RhythmID correlation coefficient is programmable is some devices by Boston Scientific. Reviewing the measured RhythmMatch values for previous episodes of VT and SVT (induced or spontaneous) facilitates programming changes. To increase the likelihood of appropriate treatment of VT, the RhythmMatch threshold should be programmed above the measured RhythmMatch values of any VTs. To increase the likelihood of appropriate inhibition of therapy for SVT, the RhythmMatch threshold should be programmed below the measured RhythmMatch values of any SVTs. In general, the sensitivity of VT detection declines with lower programmed RhythmMatch threshold values (Fig. 5). Therefore, for maximum sensitivity to VT, the highest appropriate RhythmMatch threshold value should be programmed. The nominal RhythmMatch value is 94 %.

## Committed shock/reconfirmation of the ventricular arrhythmia

Committed shock/reconfirmation refers to the monitoring performed by the pulse generator before delivery of a ventricular shock. Ventricular shock therapy can be programmed as committed or noncommitted.

## Automatic gain control

**Fig. 6:**
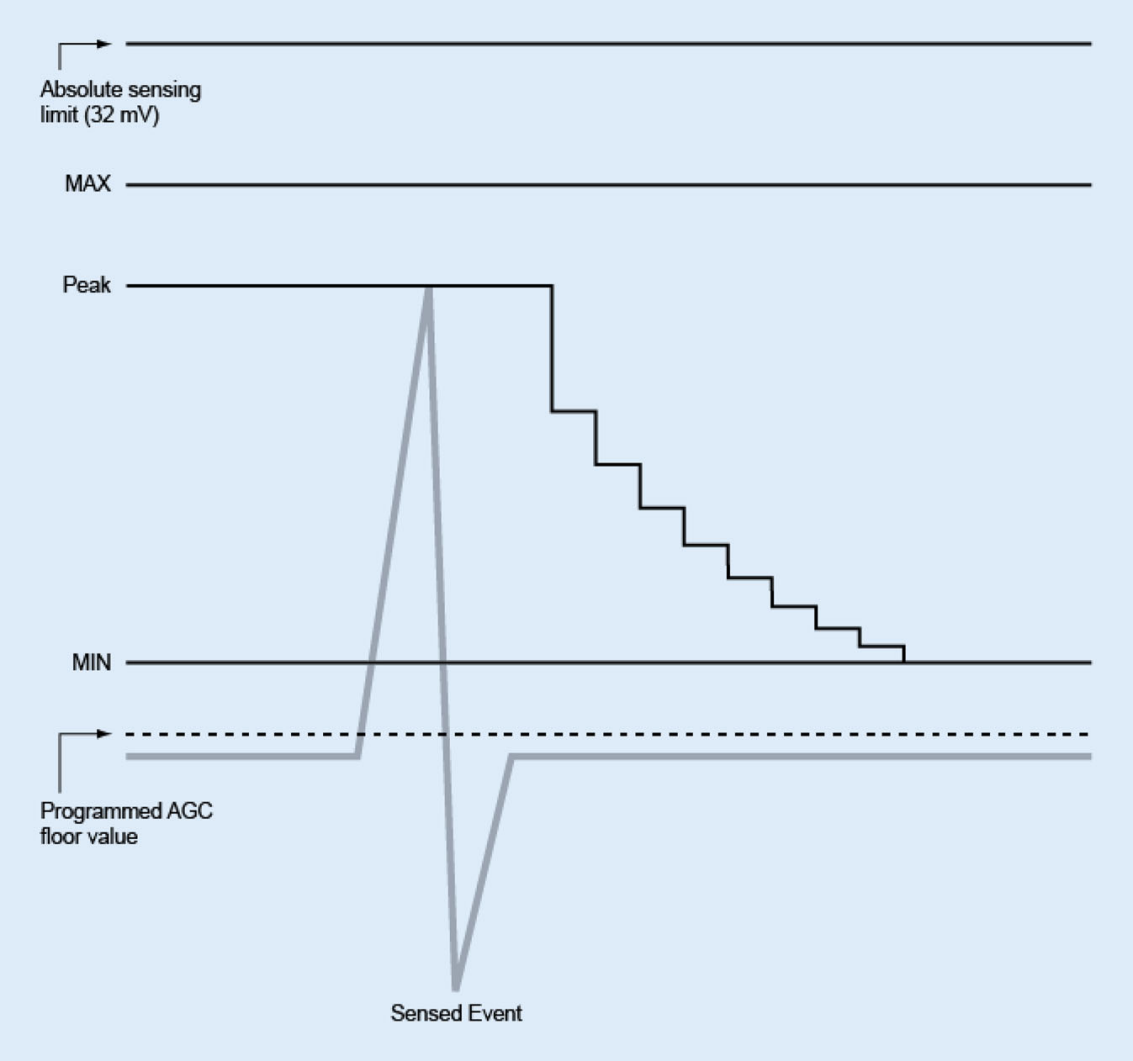
Automatic gain control (*AGC*) sensing

The pulse generator uses digital automatic gain control (AGC) to dynamically adjust the sensitivity in both the atrium and the ventricle. The pulse generator has independent AGC circuits for each chamber. Cardiac signals can vary widely in size and rate. The programmable AGC value is the minimum sensitivity value (floor) that could be reached between one beat and the next beat. This programmable value is not a fixed value present throughout the cardiac cycle; rather, the sensitivity level begins at a higher value (based on the peak of a sensed event or a fixed value for a paced event) with decrements toward the programmed floor (Fig. 6).

## Dynamic noise algorithm

The nonprogrammable dynamic noise algorithm is active in rate channels where AGC sensing is used. The dynamic noise algorithm is a separate noise channel for each chamber that continuously measures the baseline signal that is present and is designed to adjust the sensitivity floor to minimize the effects of noise. The algorithm uses the characteristics of a signal (frequency and energy) to classify it as noise. When persistent noise is present, the algorithm is designed to minimize its impact, which may help to prevent oversensing myopotentials and the associated inhibition of pacing. Noise that affects the sensing floor may be visible on the intracardiac EGMs, but would not be marked as sensed beats. However, if the noise is significant, the floor may rise to a level above the intrinsic EGM and the programmed noise response behavior (asynchronous pacing or inhibit pacing) will occur.

## Detection and automatic gain control

The right ventricular AGC is set to a nominal value of 0.6 mV and can be adjusted using the programmer. Adjustment of the AGC may be considered for cases with low-amplitude EGMs and delay in time to therapy. Any adjustment of the AGC must be evaluated in combination with the programmed detection rate thresholds/zones to ensure appropriate rate detection of the expected tachyarrhythmia. The AGC may not reach its programmed floor when tachyarrhythmia detection rates are rapid and the arrhythmia is polymorphic. Inspection of the stored EGMs and rate plots, with use of the on-screen calipers for EGM amplitude and timing measurement, permits the physician to interpret whether there are ventricular beats that are not detected. If there are unmarked beats, then an assessment should be made to determine whether programming slower rate zones would improve detection and facilitate the overall detection behavior.

## Device programming considerations

The appropriate number of therapy zones (VT-1, VT, VF) should be selected to treat the expected VTs based on the tachyarrhythmia hemodynamic stability, patient indications, and the individual patient’s clinical characteristics. This will determine the number of detection zones. Device settings for ICDs and CRT-Ds can be found in Appendix B of the HRS/EHRA ICD programming recommendations by Wilkoff et al. [3].

**Table 5:** MADIT-RIT programmed settings used during the study

MADIT-RIT three treatment arms (abbreviated)		
Arm A (conventional)	Arm B (high rate)	Arm C (duration-delay)
*Zone 1* ≥170 bpm, 2.5 s delay Onset, stability, detection enhancements on ATP + shock SRD ≥ minimum initial	*Zone 1* 170 bpm Monitor only	*Zone 1* ≥170 bpm, 60 s delay RhythmID® detection enhancement on ATP + shock SRD Off
*Zone 2* ≥200 bpm, 1 s delay Quick Convert™, ATP Shock	*Zone 2* ≥200 bpm, 2.5 s delay Quick Convert™, ATP, shock	*Zone 2* ≥200 bpm, 12 s delay RhythmID® detection enhancement on ATP + shock SRD on
		*Zone 3* ≥250 bpm, 2.5 s delay Quick Convert™, ATP, shock

*ATP* antitachycardia pacing therapy, *SRD* sustained rate duration

For patients with a primary prevention indication, MADIT-RIT Arm B or C (Table 5) programming should be considered, since these are the settings for Boston Scientific devices studied in a prospective randomized trial and proven to be superior to conventional programming for reduction of inappropriate therapies. It should be noted that the MADIT-RIT trial suggests that there may be a potential mortality benefit with Arm B programming.

Patients with secondary prevention indications were not studied in MADIT-RIT and for these patients individualized programming is recommended.

To provide sufficient opportunity for detection, the rate threshold value(s) should be programmed at least 10 min−1 below the rate of known arrhythmia(s) intended to be treated. The device detection and subsequent therapy may be different for the same underlying tachyarrhythmia depending on the number of zones and programmed parameters such as rate threshold, detection time, and detection enhancements. Boston Scientific funded the MADIT-RIT Clinical Trial [4].

## Stored EGMs with annotated markers

The pulse generator can store annotated EGMs sensed from the following sources: shock, RV atrial pace/sense lead(s) prior to the onset of an episode, around duration met, and around therapy start and end. The sensing and storage design concept allows the user to evaluate stored EGMs by using the electronic caliper (slider bar) to measure the distance/time between signals as well as to measure the amplitude of signals. The distance between signals can be measured by moving each caliper to the desired points on the EGM. The time (in milliseconds or seconds) between the two calipers will be displayed. The amplitude of the signal can be measured by moving the left-hand caliper over the peak of the desired signal. The value (in millivolts) of the signal will be displayed on the left side of the EGM. The signal is measured from baseline to peak, either positive or negative.

In CRT-D/CRT-P devices with an active LV lead, real-time LV EGMs can be used to assess LV lead performance and to assist in optimizing some programmable parameters (e. g., AV delay, LV offset). LV EGMs and associated LV event markers are available for display or printing in all sense configurations.

## Conclusion

Programming of ICDs may affect patient outcomes. Understanding of the device behavior is important when programming cardiac implantable electrical devices (CIEDs). Boston Scientific system references and physician technical manuls are intended to provide information on features and functions for implant and follow-up procedures. The Boston Scientific S‑ICD concept and algorithms are different from transvenous ICD/CRT-Ds and not included in this manuscript. Please consult Boston Scientific for additional information if needed.
